# Rapid and accurate assessment of GPCR–ligand interactions Using the fragment molecular orbital‐based density‐functional tight‐binding method

**DOI:** 10.1002/jcc.24850

**Published:** 2017-07-04

**Authors:** Inaki Morao, Dmitri G. Fedorov, Roger Robinson, Michelle Southey, Andrea Townsend‐Nicholson, Mike J. Bodkin, Alexander Heifetz

**Affiliations:** ^1^ Computational Chemistry Evotec (UK) Ltd 114 Innovation Drive, Milton Park Abingdon Oxfordshire OX14 4RZ United Kingdom; ^2^ Research Center for Computational Design of Advanced Functional Materials (CD-FMat) National Institute of Advanced Industrial Science and Technology (AIST) 1‐1‐1 Umezono Tsukuba Ibaraki 305‐8568 Japan; ^3^ Institute of Structural & Molecular Biology, Research Department of Structural & Molecular Biology, Division of Biosciences University College London London WC1E 6BT United Kingdom

**Keywords:** drug discovery, protein, *ab initio*, DFTB, MP2, GPCR

## Abstract

The reliable and precise evaluation of receptor–ligand interactions and pair‐interaction energy is an essential element of rational drug design. While quantum mechanical (QM) methods have been a promising means by which to achieve this, traditional QM is not applicable for large biological systems due to its high computational cost. Here, the fragment molecular orbital (FMO) method has been used to accelerate QM calculations, and by combining FMO with the density‐functional tight‐binding (DFTB) method we are able to decrease computational cost 1000 times, achieving results in seconds, instead of hours. We have applied FMO‐DFTB to three different GPCR–ligand systems. Our results correlate well with site directed mutagenesis data and findings presented in the published literature, demonstrating that FMO‐DFTB is a rapid and accurate means of GPCR–ligand interactions. © 2017 Authors. Journal of Computational Chemistry Published by Wiley Periodicals, Inc.

## Introduction

The rationalization of potency and selectivity in the drug discovery process requires an accurate understanding of the binding interactions between a protein and its ligand.^1^ However, visual inspection and force‐field‐based molecular mechanics calculations (MM) cannot always explain the full complexity of the molecular interactions, in particular CH‐π, halogen‐π, cation‐π, and nonclassical H‐bonds, that play critical roles in receptor–ligand binding.^2^ The use of quantum mechanical (QM) methods can take into account charge fluctuations and dynamic polarization, which are essential in assessing molecular interactions. However, despite the many advantages that QM can bring, traditional QM methods are not feasible for large biological systems, such as proteins, due to their high computational cost.

The FMO method^3^ accelerates traditional QM methods, by dividing the system into smaller pieces called fragments and performing QM calculations on these fragments (Supporting Information Fig. S1). FMO can be combined^4^, ^5^ with a fast QM method, density‐functional tight‐binding (DFTB) approach.^6^ A key advantage of FMO is that it can provide the individual contribution of each residue–ligand pair interaction energy (PIE) to the total interaction energy (TIE). TIE is a sum of PIEs for all residues; it is an estimate of the total protein‐ligand binding energy; whereas PIEs are residue contributions to it.

G‐protein coupled receptors (GPCRs) are a large and well‐studied family of membrane proteins that comprise the targets for about 30% of all pharmaceuticals currently on the market.^7^, ^8^ There are over 800 GPCR proteins encoded in the human genome, but drugs have only been developed against <10% of these targets. Thus, there is huge potential to expand the number of targets for which new therapies can be developed. To develop new drugs, both for novel and for existing targets, it is essential to understand at a molecular level the interactions that take place between ligand and GPCR.

We previously illustrated^2^ how the FMO‐MP2 method can be applied to several Class A GPCR–ligand crystal structures to explore receptor–ligand interactions. In this communication, we have extend our studies of receptor–ligand interactions by selecting three of these cases and using them to establish the reliability, speed and utility of FMO‐DFTB in comparison with FMO‐MP2. MP2 is thus used as an established reference for validating DFTB. DFTB is based on a series expansion of electron density and, as such, can be considered an approximation to density functional theory (DFT).^6^ The cost of performing MP2 calculations of fragments scales as *N* ^5^, where *N* is the number of basis functions in the fragment, due to the transformation of two‐electron integrals. In contrast, the cost of DFTB is *N* ^3^, two orders of magnitude lower, due to the Fock matrix diagonalization. Further, MP2 requires an expensive calculation of two‐electron integrals, and the cost of assembling the Fock matrix in DFTB is low because it is parametrized. Finally, the FMO‐specific electrostatic embedding in DFTB uses point charges whereas electron densities are used in MP2 calculations.

The three reference systems for this comparison are: (1) BI167107 in complex with the human β_2_‐Adrenoceptor (PDB entry 3SN6)^9^; (2) JDTic in complex with the κ‐opioid receptor (PDB entry 4DJH)^10^; and, (3) AZD1283 in complex with the human P2Y12 receptor (PDB entry 4NTJ).^11^ We selected these systems because of the extensive structure activity relationship (SAR) data available in the literature for these ligands (Supporting Information Tables S2–S4). These analogs of the crystal ligands were docked into the relevant receptors as described previously^2^ and the TIEs calculated by FMO‐DFTB.

By performing calculations on a PC cluster with 32 CPU cores, we have determined that FMO‐DFTB is approximately 1000 times faster than the standard FMO‐MP2 approach (Supporting Information Table S1). FMO‐DFTB calculations can be performed in seconds, being practical for drug discovery projects.

**Figure 1:**
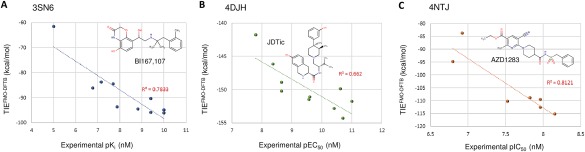
Correlation plots between experimentally measured affinity and TIE^FMO^ ^‐DFTB^ for 3 systems: a) 3SN6, b) 4DJH, and c) 4NTJ. Computationally obtained values are shown on the *y*‐axis and experimental values are shown on the *x*‐axis. [Color figure can be viewed at wileyonlinelibrary.com]

**Figure 2:**
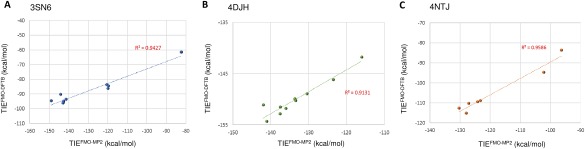
Correlation plots between TIE^FMO^ ^‐MP2^ (shown on the *x*‐axis) and TIE^FMO^ ^‐DFTB^ (shown on the *y*‐axis) for the three systems: a) 3SN6, b) 4DJH, and c) 4NTJ. [Color figure can be viewed at wileyonlinelibrary.com]

The TIEs values calculated by FMO‐DFTB can be compared with the experimental ligand binding affinities (Fig. 1) and with TIEs calculated with MP2 (Fig. 2). We observed a significant correlation (*r* ^2^ >0.66) between the calculated (TIE^FMO^ ^‐DFTB^) and the experimental values (Fig. 1). For 3SN6 and 4NTJ, we observed a significant correlation (*r* ^2^ >0.78) between the experimental values and the corresponding TIEs. In the case of 4DJH, the correlation was lower (*r* ^2^=0.66) and may have arisen as a consequence of the large error margins observed in the experimentally measured data, as reported in the literature. The high correlation between calculated and experimental values demonstrates that FMO‐DFTB provides a realistic assessment of TIE and offers additional insight into structure‐based drug design for GPCR targets.

The TIE values computed using FMO‐DFTB are in excellent agreement (*r* ^2^ >0.90) with the corresponding values calculated using FMO‐MP2 (Fig. 2), demonstrating that the performance of FMO is not compromised by the speed obtained with FMO‐DFTB. To elaborate the comparison, atomic charges in MP2 and DFTB are plotted in Supporting Information Figure S2. The good correlation implies that the electrostatic interaction (a part of PIE) in the two methods also correlates well; consistently with the total PIEs, the electrostatic contribution, based on atomic charges, is smaller in DFTB compared to MP2.

**Figure 3:**
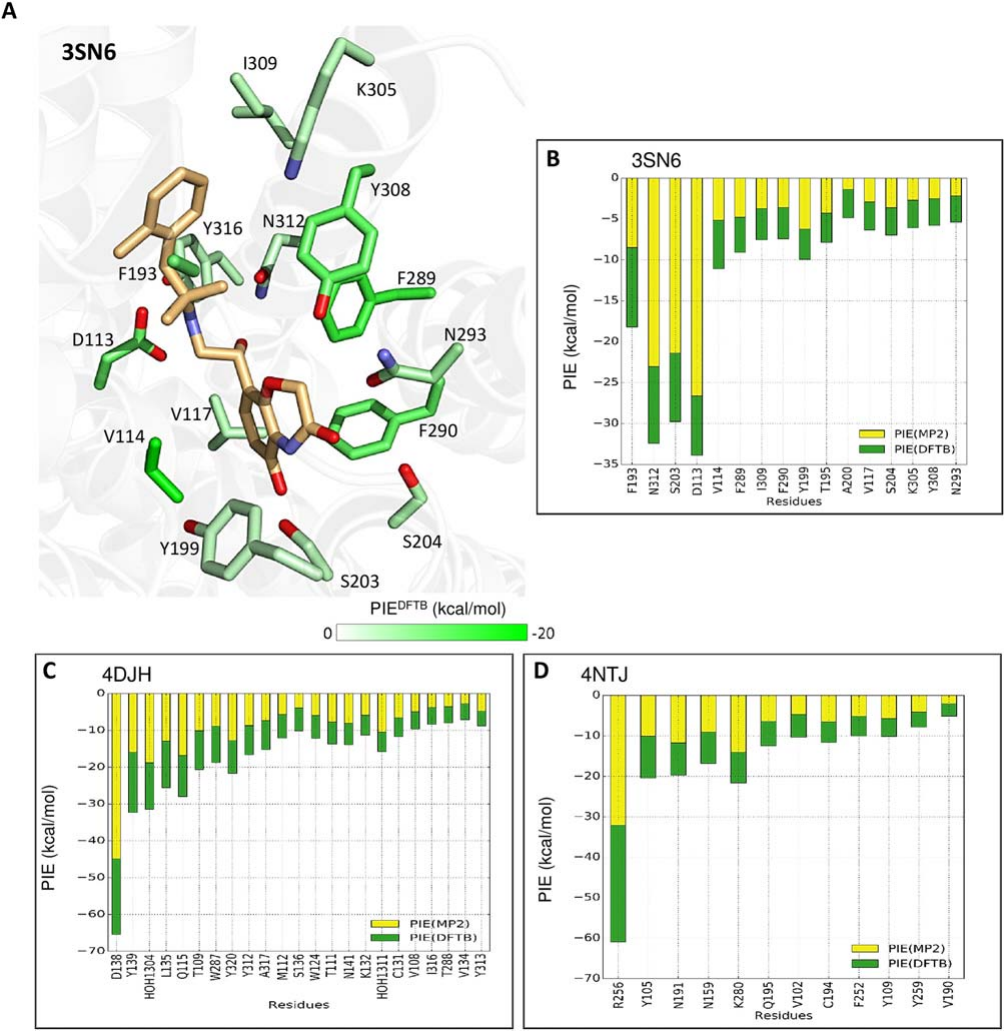
FMO‐DFTB results for (a) the human β_2_‐Adrenoceptor in complex with BI167107 (PDB entry 3SN6). The carbon atoms of the ligand are shown in light orange and the receptor residues are colored according to the PIE values calculated by FMO‐DFTB (shown by the PIE^DFTB^ bar in the lower right hand corner of the panel. Nitrogen atoms are shown in blue, oxygen in red, sulphur in yellow and chlorine in light green. Sorted PIE values for GPCR residues calculated at DFTB and MP2 levels (in green and yellow, respectively) are shown for the β2‐adrenoceptor (b), the κ‐opioid receptor in complex with JDTic (c), and the human P2Y_12_ receptor in complex with ASD1283 (d). [Color figure can be viewed at wileyonlinelibrary.com]

We used FMO‐DFTB to calculate individual residue–ligand pair interaction energies (PIEs) for the three systems (Fig. 3). We consider any interaction with an absolute PIE ≥ 3.0 kcal/mol to be significant. The water molecules in this work were extracted from the crystal structures (if resolved) and treated explicitly to explore their contribution to the receptor–ligand binding.

β_2_‐Adrenoceptor receptor (β2AR) is primarily located in the heart and the kidney, where it is involved in physiological processes including the regulation of heart rate and blood pressure. This first case was used to illustrate how the FMO‐DFTB results can be visualised, namely as a 3D figure (Fig. 3a) or with the data displayed in a plot (Fig. 3b). FMO‐DFTB detected 17 significant interactions in this system. The majority of these interactions are consistent with literature reports^9^ and with those calculated at MP2 level.^2^ Novel interactions with residues Val114 and Lys305 have been identified using FMO‐DFTB. While no information is available for Lys305, the mutation from valine to alanine at Val114 has been reported^9^ to disrupt the binding of agonists and antagonists. According to this analysis, Val114 forms unusual CH‐π bonds.^12^ This result is in agreement with our previous findings^2^ where we extensively explored the role of nonclassical interactions, such as CH‐π, in GPCR–ligand binding.

The opioid system controls pain, as well as reward and addictive behaviours. Opioids exert their pharmacological actions through activation of the three opioid receptors, μ (MOR), δ (DOR), and k (KOR). JDTic is a long‐acting (“inactivating”) antagonist of the KOR, and is highly selective for the μ‐ and δ‐ opioid receptors and nociceptin receptor. The JDTic‐KOR complex is the first crystal structure of an opioid receptor.^10^ FMO‐DFTB calculations highlighted 23 strong interactions (Fig. 3c). These interactions correlate well with the literature, where the selectivity of JDTic has been rationalized by interactions with residues Val108 and Tyr312.^13^ In addition, FMO‐DFTB identified much stronger interactions than those in the literature,^10^ including that of Asp138, highlighting the utility of this method for predicting residues for further experimental study.

The P2Y_12_ receptor is considered to be one of the most promising drug targets for antiplatelet therapies. AZD1283 is a novel P2Y_12_ antagonist for the treatment of arterial thrombosis and was recently progressed into human clinical trials. FMO‐DFTB identified 12 relevant interactions for this system (Fig. 3d). These results are consistent with previous experimental findings.^11^ The strongest interaction identified is at Arg256, which is a residue shown to interact with non‐nucleotide antagonists.^14^ Interestingly, P2Y_12_ receptor signalling has been shown to be impaired in a patient with an Arg to Gln mutation at position 256.^15^

We have demonstrated that FMO‐DFTB is a rapid, accurate and reliable method for the assessment of receptor–ligand interactions and TIE calculations. The interactions detected by FMO‐DFTB are consistent with the experimental data and with those detected by FMO‐MP2.^2^ The application of FMO‐DFTB will be of great utility for the design and evaluation of new compounds, providing a means of significantly decreasing the effort and cost of chemical synthesis needed for drug discovery programs.^16^ The high correlation between receptor‐ligand experimentally evaluated affinity and TIE^FMO^ ^‐DFTB^ indicates that FMO‐DFTB can be used to determine the binding affinities of new targets and, therefore, provides a means of accurately predicting experimental outcomes.

For the first time, it is now possible to perform QM calculations for protein‐ligand complexes in a high throughput manner to address the needs of processing large amounts of SAR data. Docked ligands can be refined, rescored, and reranked with FMO‐DFTB in the presence of the surrounded protein and water molecules. In summary, FMO‐DFTB possesses the accuracy of much more expensive methods (FMO‐MP2) at a dramatically enhanced speed, making it a very attractive method to support rational SBDD against GPCR and other drug targets.

## Computational Methods

We applied FMO code^17^ version 5.1 distributed inside *ab initio* quantum chemistry package GAMESS.^18^ We used the third order DFTB3 method^6^ with 3ob parameters,^19^, ^20^ and the Møller–Plesset second order perturbation theory (MP2) and, for treating solvent effects, we combined both calculations with the polarizable continuum model (PCM).^4^ MP2 was used with the 6–31G* basis set whereas the UFF dispersion model was used for DFTB3. The structures were taken from the previous study.^2^

## Supporting information

Supporting Information
