# Supplementary material for: Rapid and accurate assessment of GPCR–ligand interactions Using the fragment molecular orbital‐based density‐functional tight‐binding method
Source: J Comput Chem. 2017 Jul 4;38(23):1987–90. doi: 10.1002/jcc.24850 (PMC5600120; doi:10.1002/jcc.24850)
Supplement: Supplementary file 1 — Supporting Information [file JCC-38-1987-s001.docx]

Rapid and accurate Assessment of GPCR-Ligand Interactions Using the Fragment Molecular Orbital Based Density-Functional Tight-Binding (FMO-DFTB) Method

*Inaki Morao^1^, Dmitri G. Fedorov^2^, Roger Robinson^1^, Michelle Southey^1^, Andrea Townsend-Nicholson^3^, Mike J. Bodkin^1^ and Alexander Heifetz^1^**

^1^Evotec (UK) Ltd., 114 Innovation Drive, Milton Park, Abingdon, Oxfordshire OX14 4RZ, United Kingdom^;^

^2^CD-FMat, National Institute of Advanced Industrial Science and Technology (AIST), 1-1-1 Umezono, Tsukuba, Ibaraki 305-8568, Japan

^3^Institute of Structural & Molecular Biology, Research Department of Structural & Molecular Biology, Division of Biosciences, University College London, London, WC1E 6BT, United Kingdom

* Corresponding authors: D. G. Fedorov: d.g.fedorov@aist.go.jp and A. Heifetz, Alexander.Heifetz@evotec.com


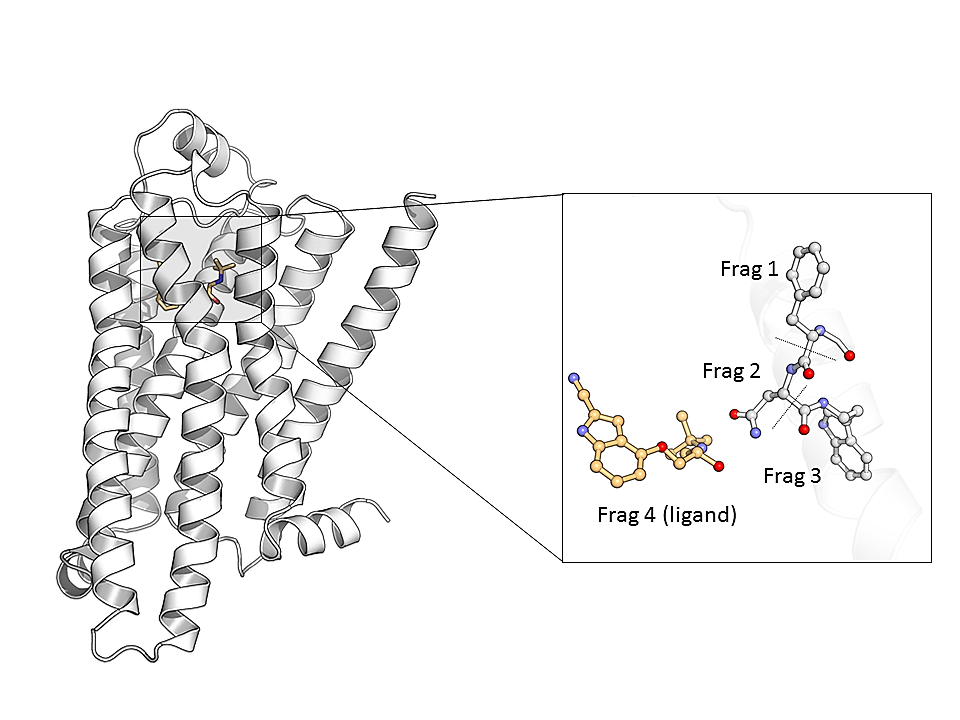


Figure S1. FMO fragmentations are achieved by dividing the receptor protein into fragments, each of which corresponds to an amino acid (Frag 1-3). Similarly, the ligand can be represented by single or multiple fragments as necessary (Frag 4).

Figure S2. Correlation of the Mulliken atomic charges in MP2 and DFTB (atomic charges in MP2 are calculated at the uncorrelated level), for 1 ligand for each protein.

**Table S1.** Comparison of FMO-MP2 and FMO-DFTB computational times in seconds for GPCR-ligand systems

|  | **FMO-MP2** | **FMO-DFTB** | **Speed-up factor** |
| --- | --- | --- | --- |
| **3SN6** | 19,458 | 12 | 1,621 |
| **4DJH** | 32,577 | 32 | 1,018 |
| **4NTJ** | 25,118 | 28 | 897 |
| **Average** | 25,717 | 24 | 1,178 |

**Table S2:** Raw data collected from the literature^3^ for the FMO SAR analysis of BI167107 analogs bound to the human β_2_-Adrenoceptor complex (PDB entry 3SN6)

| **Structure** | **ID** | **pEC50** |
| --- | --- | --- |
|  | BI167107 | 10.00 |
|  | 6c | 9.40 |
|  | 6d | 10.00 |
|  | 6e | 8.82 |
|  | 6f | 7.87 |
|  | 6i | 9.40 |
|  | 8a | 7.15 |
|  | 8b | 7.70 |
|  | 8d | 6.77 |
|  | 10d | 5.00 |

**Table S3:** Raw data collected from the literature^1^ for the FMO SAR analysis of JDTic analogs bound to the human κ-opioid receptor (PDB entry 4DJH)

| **Structure** | **ID** | **pKe** |
| --- | --- | --- |
|  | JDTic | 10.70 |
|  | 4 | 10.62 |
|  | 5 | 11.00 |
|  | 6 | 10.41 |
|  | 7 | 9.57 |
|  | 8 | 9.60 |
|  | 9 | 8.37 |
|  | 10 | 8.66 |
|  | 11 | 7.80 |
|  | 12 | 8.65 |

**Table S4:** Raw data collected from the literature^2^ for the FMO SAR analysis of AZD1283 analogs bound to the human P2Y_12_ receptor (PDB entry 4NTJ)

| **Structure** | **ID** | **pIC50** |
| --- | --- | --- |
|  | AZD1283 | 7.96 |
|  | 24 | 6.92 |
|  | 25 | 7.82 |
|  | 26 | 7.52 |
|  | 27 | 8.15 |
|  | 28 | 6.80 |
|  | 29 | 7.96 |

1. Kormos, C. M.; Gichinga, M. G.; Maitra, R.; Runyon, S. P.; Thomas, J. B.; Brieaddy, L. E.; Mascarella, S. W.; Navarro, H. A.; Carroll, F. I., Design, synthesis, and biological evaluation of (3R)-1,2,3,4-tetrahydro-7-hydroxy-N-[(1S)-1-[[(3R,4R)-4-(3-hydroxyphenyl)-3,4-dim ethyl-1-piperidinyl]methyl]-2-methylpropyl]-3-isoquinolinecarboxamide (JDTic) analogues: in vitro pharmacology and ADME profile. *J Med Chem* **2014**, 57, 7367-81.

2. Bach, P.; Antonsson, T.; Bylund, R.; Bjorkman, J. A.; Osterlund, K.; Giordanetto, F.; van Giezen, J. J.; Andersen, S. M.; Zachrisson, H.; Zetterberg, F., Lead optimization of ethyl 6-aminonicotinate acyl sulfonamides as antagonists of the P2Y12 receptor. separation of the antithrombotic effect and bleeding for candidate drug AZD1283. *J Med Chem* **2013**, 56, 7015-24.

3. Hoenke, C.; Bouyssou, T.; Tautermann, C. S.; Rudolf, K.; Schnapp, A.; Konetzki, I., Use of 5-hydroxy-4H-benzo[1,4]oxazin-3-ones as beta2-adrenoceptor agonists. *Bioorg Med Chem Lett* **2009**, 19, 6640-4.
